# Participation to Leisure Activities and Well-Being in a Group of Residents of Naples-Italy: The Role of Resilience

**DOI:** 10.3390/ijerph17061895

**Published:** 2020-03-14

**Authors:** Sergio Cocozza, Pier Luigi Sacco, Giuseppe Matarese, Gayle D. Maffulli, Nicola Maffulli, Donatella Tramontano

**Affiliations:** 1 Department of Molecular Medicine and Medical Biotechnology, University “Federico II” of Naples, 80131 Naples, Italy; sergio.cocozza@unina.it (S.C.); giuseppe.matarese@unina.it (G.M.); 2 Department of Humanities, IULM University Milan, 20143 Milan, Italy; 3 metaLAB (at) Harvard and Berkman-Klein Center for Internet & Society, Harvard University, Boston, MA 02138, USA; 4 Bruno Kessler Foundation, 38122 Trento, Italy; 5 Centre for Sports and Exercise Medicine, Barts and The London School of Medicine and Dentistry, Mile End Hospital, 275 Bancroft Road, London E1 4DG, England; g.maffulli@qmul.ac.uk (G.D.M.); nmaffulli@unisa.it (N.M.); 6 Department of Musculoskeletal Surgery, University of Salerno School of Medicine, Surgery and Dentistry, 84121 Salerno, Italy

**Keywords:** subjective well-being, physical activity, cultural participation, resilience, Naples

## Abstract

We explored the relationship between cultural and social participation, physical activity, and well-being in a group of residents of the metropolitan area of Naples, Italy and the role that resilience plays in this relationship. Naples offers a remarkable urban environment with the potentially beneficial psychological effects of outstanding natural beauty, and one of the world’s most impressive repositories of tangible and intangible cultural heritage. However, Naples was also, and still is, heavily affected by the 2008 economic crisis, in addition to preexisting social and economic issues. The major finding of this study is that, despite this highly contrasting urban environment, the combination of physical activity and engagement in social and cultural activities has a positive effect on subjective (self-reported) psychological well-being (SPWB) in a group of residents, and that resilience mediates this relationship.

## 1. Introduction: Naples as a Unique Urban Environment 

### 1.1. The Context of the Study: A City of Contrasts, Juxtaposing Deep-Seated Cultural Vibrancy and Socio-Economic Dysfunction 

The city of Naples is an interesting model of urban contrasts. Naples is located on the Bay of Naples overlooking the Tyrrhenian Sea and Capri and Ischia Islands, also sitting between two of the most dangerous volcanoes in the world: Mount Vesuvius and the Phlegraean Fields. Mount Vesuvius, best known for its eruption in AD 79 that led to the burying and destruction of Pompeii and Herculaneum, erupted at least fifty times thereafter, most recently in 1944. The Phlegraean Fields are a vast and restless volcanic cauldron covering a 100 km^2^ area, and Europe’s only super-volcano. A super-volcano is a volcano which had in the past an eruption of magnitude 8—the highest and most violent possible—on the volcano explosivity Index. The Phlegraean Fields owe their formation, some 39,000 years ago, to the most violent eruption in the past 200,000 years in Europe. The Phlegraean Fields last erupted in 1538. Although it was only a minor eruption, it was the origin of Monte Nuovo, the youngest mountain in Europe. Today, this area is one of the most dangerous volcanic regions in the world, as about 4.5 million people live nearby. 

The recorded history of Naples begins in the 7th century BC. The city was ruled by Greeks, Romans, Ostrogoths, Byzantines, Lombards, Normans, Hohenstaufens (the powerful Germanic royal house of Swabian origin), Angevins, Spanish Aragonese, the Habsburg and Bourbon families, Austrian, French (under Napoleon), and finally joined the Kingdom of Italy in 1861 [1]. Archeological ruins, historical buildings, royal palaces, fortified castles, (more than five hundred) churches and museums still testify the diverse cultures that have contributed to shape the city and its unique urban environment in the course of three thousand years. In 1995 UNESCO listed Naples’ historic city center as a World Heritage Site, “because of its remarkable urban and natural environment, and one of the world’s most impressive repositories of tangible and intangible cultural heritage” [2]. 

Once it joined the Kingdom of Italy, the city entered a time of decline that profoundly marked its urban culture and inhabitants. A yearning for lost grandeur, a mourning for what cannot be retrieved, and an internalized feeling of resignation, and sometimes resentment, towards the uncontrollable external forces that have undermined the city’s greatness are still strongly rooted in the city’s collective imaginary. A poignant literary rendition of this psychological condition, among others, is offered by the collection of short stories by Anna Maria Ortese, “Il mare non bagna Napoli” (The sea does not touch Naples) [3]. After national unification, the economy collapsed and, between 1876 and 1913, in an unprecedented wave of emigration, at least four million of citizens of Naples and the surrounding areas left for the North and abroad. It was the beginning of the still standing “North-South divide”, that is the economic gap between the rich industrialized North and the poor, more agrarian South of the country. A heated debate has engaged economists, sociologists, and historians for decades as to the key reasons for the North-South divide. Francesco Saverio Nitti (1868–1953), a major economist and political figure of the time, stated in his book “Il bilancio dello Stato dal 1862 al 1897” [4] that the contribution of the Kingdom of the Two Sicilies to the finances of the new, unified state was one of “less debts and huge public richness”, and moreover soon after unification ”there was a flow of richness from South to North”: 80 million ducats were taken from the wealthy Bourbon Kingdom banks as a contribution to the new Italian treasury. Whether the divide preexisted the unification or was caused by it, however, is still an open issue. As a matter of fact, the North-South gap in terms of GDP per capita, industrialization, infrastructural endowment and socio-economic development is ample, well documented, and persistent [5,6].

As a consequence, the 2008 worldwide recession affected Naples, the largest city of the Italian South, more than other area of the country. After 2008, a number of studies reported the negative impact of the fall in local GDP, increased unemployment, and banking crashes on the psychological well-being of residents [7,8,9,10]. In addition to the economic downturn, the Naples metropolitan area is still paying the toll of the 1980 earthquake. The post-disaster re-construction basically resulted in a huge building speculation which heavily affected the quality of Naples’ built environment in a negative sense, as was already the case in the 1886–1913 building cycle, and in the post-WWII period of the “Italian economic miracle”. In particular, in the 1980s uncontrolled overbuilding devastated Naples’ suburbs, which soon after suffered from a massive takeover of organized crime clans, with explosive consequences on the breakdown of the social fabric, not only in the suburbs themselves, but also across the whole city, further exacerbating social and health inequalities. Such a dysfunctional urban environment, built as we have seen through a centuries-long stratification of negative circumstances, is a potentially pathogenic socio-economic context in terms of both physical and mental health, inducing unhealthy lifestyles (physical inactivity, unhealthy diets, tobacco smoking, harmful use of alcohol, among others [11,12]); and magnifying the negative effects of social isolation. The impact of social isolation on risk of death and diseases, such as cardiovascular ones, is in turn comparable to well-established risk factors such as smoking and alcohol consumption, and exceeds that of risk factors such as physical inactivity and obesity [13,14,15]. 

Despite the difficult local conditions, Naples kept attracting people from all over the world across the centuries, including creative geniuses such as Boccaccio, Caravaggio, Mozart, Dumas, Goethe, Gorky, Warhol, and Kounellis, to name a few. In spite of the dysfunctional urban environment, Naples possesses a widely recognized charm in which natural beauty, richness of its cultural heritage, food culture, and artistic and musical vitality stand on a par with other world-celebrated cities such as Rio de Janeiro and New Orleans. A vibrant local community of artists, writers, gallery owners, film directors, and actors, most of whom are well known worldwide, work tirelessly to keep Naples’ cultural life alive and open to cutting-edge creation and experimentation. The vitality of the cultural life of Naples relies also on five prestigious Universities, among which the Federico II University of Naples, founded in 1227 by the Emperor Frederick II, is the oldest public university in the world. In the city are located several research centers whose contribution to biomedicine, engineering, aero spatial science, volcanology, marine biology are internationally recognized. In 1872, Anton Dohrn funded the Zoological Station in Naples, the first marine biology center in the world exclusively dedicated to research and advanced teaching. 

To our knowledge, the effects on psychological well-being of this kind of unique, culturally rich urban environments characterized by a long, troubled history but also by an exceptionally stimulating sensory, aesthetic and emotional cultural ecosystem, have not yet been studied systematically. However, the peculiarity of such environments may be very helpful in improving our understanding of the factors that promote psychological well-being. The urban environments that generally sit in high positions in world rankings of quality of life, and are therefore legitimized as good practices to orientate and inspire city planners worldwide, tend to be highly functional, well-organized ones, whereas cities like Naples fare poorly. But cultural charm and vibrancy are elusive concepts that do not simply boil down to, say, the quality and variety of local cultural events. Therefore, it is legitimate to wonder to what extent such definitions of ‘quality of life’ really reflect all the dimensions that make living in a city stimulating and enjoyable. Clearly, the effect of the urban environment, and of its cultural richness more specifically, on subjective well-being should matter in the definition of what we mean by quality of life. With this study, we want to investigate this overlooked point as part of a more general reflection on the relationship between heritage cities and well-being. 

### 1.2. Culture and Psychological Well-Being in Dysfunctional Urban Environments: A Neglected Possibility?

The previous discussion illustrates why Naples is a case of special interest in its dramatic contraposition of socio-economic dysfunctionality and artistic and creative excellence, which is deeply rooted into the city’s popular culture and not only the elite one. It is therefore natural to ask to what extent this contraposition reflects into the perceptions of well-being of residents, and whether the unique socio-cultural circumstances of the city provide a counter-balancing effect to the negative impact of other environmental factors, also in terms of resilience to urban hardships. This research question also presents potentially interesting implications for other urban environments characterized by similar contrasts, and highlights the so far mostly disregarded role of local culture as a basis for innovative public health interventions in otherwise dysfunctional urban environments.

For these reasons, we focus on leisure as the experiential dimension that enables residents to best take advantage of the city’s cultural vibrancy and natural beauty, and which potentially contributes as a positive environmental component to residents’ perceptions of well-being. Moreover, as it is commonly maintained that Neapolitan residents have developed local adaptive strategies of their own to cope with the challenging urban environment they have to deal with on a daily basis, we ask whether resilience functions as an effective mediator of the relationship between leisure and well-being. If socially relevant forms of environmental adaptation have occurred, they should in principle be traceable in terms of locally specific forms of urban resilience.

Well-being and resilience are important features for maintaining normal human functioning, in particular when facing traumatic and stressful life events. Well-being is a dynamic construct that builds upon several social, psychological, and physical dimensions, such as life satisfaction (evaluative well-being), social and family relationships, social roles and activities, sense of purpose and meaning in life (eudemonic well-being), and feelings of happiness, sadness, anger, stress, and pain (hedonic well-being) [16,17,18]. Resilience is a major component of well-being, and, although its definition has evolved over time, it is usually cast in terms of the ability of individuals to tackle life’s challenges, and to carry on and persevere in the face of adversity, even to the point of turning it into a functional development [19,20,21,22]. Since the publication in 2009 of the Report of the Stiglitz-Sen-Fitoussi Commission for the measurement of economic performance and social progress, leisure activities are included as a key indicator in all major measures and indexes of subjective well-being, life satisfaction, and happiness [23,24,25,26].

Leisure time provides people with a sense of fulfilment and personal autonomy, enables them to feel positive emotions, and to acquire valuable skills and knowledge [27]. Participation in leisure activities creates opportunities for socialization and contributes to social cohesion, by allowing people to connect to and network with others [28]. Leisure also provides opportunities to promote life balance, facilitating improved coping with daily life stress [29,30,31]. The physical activity component of leisure, in turn, is significantly associated with pursuit and maintenance of personal health [32], health-related quality of life [33,34], and psychological well-being [35], and is actively promoted as a good habit among healthy adults [36]. 

In this regard, focusing on leisure as a determinant of psychological well-being, and consequently as a source of health benefits, paves the way to possible new approaches to public policy. Even more so in public health, where leisure plays a more central and active role than commonly maintained, and particularly in a peculiar, and potentially critical, urban environment such as Naples. In this paper, we explore the association between leisure under the form of cultural, social, physical activity and well-being in a group of residents of the metropolitan area of Naples, assessing the role of resilience as a mediating factor in such relationship. 

## 2. Methods

### 2.1. Basic Features

Within the framework of the European Innovation Partnership on Active and Healthy Ageing, Action 3—Getting to Optimize Aging Life Quality (GOAL) project, Fondazione GENS Onlus (a non-profit organization; Gene Environment Interaction Studies [37]) developed an anonymous questionnaire to assess the modes and frequency of access to leisure experiences, and their relationship with perceived well-being, resilience, and perceived health. 

The questionnaire used in the present study is anonymous, in that no information was collected that could serve in any possible way to identify the participants (name, surname, address, postal code, telephone or mobile phone number, e-mail date of birth, income information). Participation in the survey was voluntary. Trained GENS personnel gave all the necessary information regarding the scope and aims of the study to the individuals willing to participate in the survey. The anonymous questionnaire (paper and pen) was handed and explained to each participant, who was requested to fill the questionnaire and hand it back to GENS personnel on the spot. On request, GENS personnel assisted participants in filling the questionnaire. In order to ensure that all the social groups would be represented in the sample, the questionnaires were randomly collected in different areas across various neighborhoods of Naples. It is important to point out that in the city of Naples, low-, middle- and high-income people typically co-habit in the same neighborhood, although in different proportions, and often even in the same building. 

In the present study, we analyze the responses of 1182 adults aged 18–93. The sample size is comparable to those of analogous studies such as the surveys on subjective psychological well-being (SPWB) carried out in Italy by Chasseny et al. in 2004, and Grossi et al. in 2011 [38,39], consisting of 1475 and 1500 subjects each, respectively. In 2012, Grossi et al. used a sample of 1000 subjects to investigate the SPWB of Milan residents [40]. 

### 2.2. Questionnaire Structure

The questionnaire used in this survey is anonymous and not anonymized. Anonymity refers to data collected from respondents who are completely unknown to anyone associated with the survey. Only the respondent knows that he or she participated in the survey, and the survey researcher cannot identify, in any possible way, the participants. No one, including the investigator, can link an individual person to the responses.

The questionnaire we collected fulfills these requirements, in that it does not contain: Name and surname, address, ZIP code, ID or social security number, contact information of any kind (phone, or e-mail address). As consequence, individuals participating in this survey cannot be discerned in any way by anyone of the researchers involved. For these reasons, anonymous surveys do not require ethical approval. In fact, in an anonymous survey, a written consent would have the paradoxical effect of compromising anonymity. Thus, the usual position in anonymous surveys is that a positive response from a respondent is, in itself, evidence of consent.

The anonymous questionnaire, in Italian, collected information covering socio-demographic and health-related data on relevant determinants of subjective well-being: 

1. *Demographic information*. Gender, age, schooling (no school, primary, secondary, high school, college), civil status (single, married, widow, divorced/separated), employment/work. 

2. *Subjective Self-reported psychological well-being (SPWB)*. Here we adopted a short form (PGWB-S) of the original psychological general well-being index [38], developed and validated in its Italian version by Grossi et al. in 2006 [41]. PGWB-S reduces the number of items from the original 22-item form of the PGWB down to 6, preserving more than 90% of the explained variance with respect to the full form, and more generally maintaining validity, reliability, and good acceptability for the use in various settings in Italy. On the other hand, PGWB-S is considerably shorter than PGWB, and this makes it much more acceptable to interviewees, and is consequently less susceptible to the biases from interviewees’ failure to comply with the demanding levels of focus and consistency required to go through the entire questionnaire. Moreover, PGWB-S calls for a shorter time of administration and presents a better response rate and a lower rate of missing data. The PGWB-S questionnaire covers the following domains: Anxiety, Vitality (positive), Depressed Mood, Self-Control, Positive Well-Being, and Vitality (negative), assessed on a 0–5 Likert scale for the four weeks before the date of the survey. For brevity, we will refer to the results of the PGWB-S questionnaire as subjective self-reported psychological well-being (SPWB).

3. *Resilience*, measured according to the two-item Connor-Davidson resilience scale (CD-RISC2) on a 0–4 Likert scale [42]. In 2005, Connor and Davidson proposed an abbreviated version of their original CD-RISC to reduce administration time. The two items used for this scale are item 1 (“Able to adapt to change”) and item 8 (“Tend to bounce back after illness or hardship”). Connor and Davidson deemed those items to be capable of “etymologically capturing the essence of resilience.” After analysis of test-retest reliability, convergent validity, and divergent validity, the CD-RISC2 demonstrated “significant correlation” with both the CD-RISC as a whole, and with the individual items of the CD-RISC. Connor-Davidson argue that since the CD-RISC2 sufficiently represents the original measure, the two-item CD-RISC2 can be utilized in place of the 25-item CD-RISC, with the advantage to reduce administration time [42]. An Italian version of CD-RISC2 was not available, and an ad hoc translation was prepared adopting the conventional forward–backward procedure [43].

4. *Cultural and social activities*. We chose, in particular, the following: movies, theater, concerts, opera and ballet, museum exhibitions, reading, dancing, social activities, and charity work. Participants had to indicate whether or not they engaged in any of these activities, and also their frequency of participation over the last year, as follows: once a week, once a month, every six months, or once a year. The list of leisure activities was prepared according to the Special Eurobarometer 399 report on cultural access and participation [44].

5. *Lifestyle habits*. Participants had to indicate whether or not they engaged in the following habits: smoking, diet, physical activity, as well as their preferred mode of transportation and patterns of computer use.

6. *Diagnosed diseases*. We considered the following list: diabetes, respiratory diseases, skin diseases, gastritis, anemia, depression, osteoporosis, kidney diseases, migraine, anxiety, heart failure, arrhythmias, ischemic heart diseases, cancer, allergy, arthritis, myocardial infarction, hypertension, obesity, liver disease, back pain, colitis, none, and other. The above reported list of diseases was prepared according to the relevant Organization for Economic Co-operation and Development (OECD) and World Health Organization (WHO) reports [45,46].

### 2.3. Statistical Strategy

Zero-order correlations among key study variables were analyzed in terms of Pearson product moment correlations (between continuous variables) and point-biserial correlations (between binary and continuous variables). Before analysis, qualitative variables were transformed in artificially dichotomized variables.

Multiple regression analysis was performed with SPWB and resilience as dependent variables. For each dependent variable, separate regression models were run for both physical activity and participation to cultural and social activities. Gender and number of diseases were included as covariates in the analyses because they showed significant zero-order correlations with both PGWB and resilience. Before performing multiple regression analysis several assumptions were tested. The linearity assumption has been tested with scatterplots. Q-Q-Plot was used to test whether the errors between observed and predicted values were normally distributed. 

Concerning multicollinearity, Pearson’s bivariate correlations among all independent variables showed correlation coefficients less than 0.80. Finally, a scatterplot of residuals versus predicted values was performed to check for homoscedasticity. 

In order to test the significance of the mediation effect, the Sobel test was used [47]. To estimate the ratio of mediated to total effect, we used the method described by Preacher and Kelley [48]. *p* values less than 0.001 were considered statistically significant. The R statistical computing environment was used for all analyses [49]. 

## 3. Results

### 3.1. Summary Statistics and Pearson’s Correlations

#### 3.1.1. Sample Description

The main characteristics of the sample population are outlined in Table 1. The sample consisted of 638 females and 544 males. The average age of the overall sample was 55.73 ± 15.70 years, whereas the range was 18–93 years. Considering that we did not collect questionnaires from participants below 18 years of age, the age distribution of the sample in our study accorded with the structure of the Neapolitan population, according to Italian Institute of Statistics. The same applies to civil status data: over 60% of the sample were married or cohabiting, while 21% were not married, and 7% were widow(er)s. About 36% of the sample had a high school education, and 8% had only an elementary school education. Retired people represent 25.5% of the sample, while 14% of the women in the sample were housewives. Unemployment was about 13%. 

The average value of PGWB score in the overall population amounted to 68.76 ± 19.18, with a range of 0–110. The average value of resilience score in the overall population was 5.601 ± 1.892.

#### 3.1.2. Health Self-Perception and Reported Diagnosed Diseases

Respondents were asked to indicate any diagnosed disease/s within the following list: respiratory diseases, skin diseases, gastritis, anemia, depression, osteoporosis, kidney diseases, migraine, anxiety, heart failure, arrhythmias, ischemic heart diseases, myocardial infarction, hypertension, diabetes, obesity, cancer, allergy, arthritis, liver disease, back pain, colitis, none, and other. In the population analyzed, the average number of reported diagnosed diseases was 2.376 ± 2.164. Cardiovascular diseases (heart failure, arrhythmias, ischemic heart diseases, myocardial infarction) were reported by 17.06% of the subjects, and their risk factors (hypertension, diabetes, obesity) by 26%, 8.75%, and 5.9%, respectively. Depression and anxiety were reported by 5.91% and 11.90%, respectively. About 62% of the subjects at the moment of the survey perceived their health as good, very good, or excellent, while 38% consider their health poor or barely acceptable. 69% of the population rated their sex life as excellent or good, while 31% rated it as barely acceptable or poor.

#### 3.1.3. Leisure Activities

84.4% of the respondents stated to be engaged in cultural and/or social activities, 82% in the male group and 86% in the female group. On average, females participate to 4.3 ± 2.5 activities with a participation index of 10.36 ± 6.4, while the male group participates to 3.8 ± 2.6 activities, with a participation index of 8.75 ± 5.8, *p* < 0.0009 and *p* < 0.0001 respectively. As for physical activity, 38% of the sample regularly exercise (39% of males and 37% of females). 

### 3.2. Pearson’s Correlations

Table 2 shows Pearson’s correlations between all the study variables. As expected, there was evidence of a statistically significant positive association between age and the number of diagnosed conditions, and of negative association with participation in cultural and social activities (*r* = −0.18, *p* < 0.001), as well as with physical activity (*r* = −0.19, *p* < 0.001). In turn, it was also expected that the number of diagnosed conditions would show evidence of negative association with both participation in social activities (*r* = −0.25, *p* < 0.001) and physical activity (*r* = −0.22, *p* < 0.001). The number of diagnosed diseases showed evidence of negative association with SPWB but not with resilience.

SPWB scores were lower in females; this finding agrees with previous studies. We also found as expected a strong association between resilience and SPWB. Finally, both participation in social activities and physical activity showed evidence of association with resilience as well as with SPWB.

### 3.3. Multiple Regression Analyses Investigating the Relationship between Participation in Cultural and Social Activities, Physical Activity and Resilience

To further analyze the relationship between participation in cultural and social activities and physical activity and resilience, we used a multiple regression approach. The aim was to separate the possible effects of the two variables on resilience. Since other variables were also significantly associated with resilience, they were included in the regression models. Two models were studied: Model 1a, which included physical activity as an independent variable, and Model 1b, which included cultural activity as an independent variable. The results of the regression analyses investigating the relationship between resilience and these two variables are shown in Table 3 and Table 4.

Both models explained a relatively low share of the total variance of resilience (respectively R-squared = 0.023 and R-squared = 0.033). It is intriguing to note that both physical and cultural activities were the independent variables most strongly related to resilience, being the only variables that reached statistical significance in the two models.

### 3.4. Multiple Regression Analyses Investigating the Relationship between Participation in Cultural and Social Activities and Physical Activity and SPWB

To explore the relationship between participation in cultural, social, and physical activity and SPWB, we used the same approach described above. Here again two models were studied: Model 2a, which included physical activity as an independent variable, and Model 2b, which included cultural activity as an independent variable. In both models, SPWB was set as a dependent variable and resilience added as an independent variable. The results are reported in Table 5 and Table 6.

Model 2a, which included resilience and physical activity as independent variables, explained 32.39% of the variance in well-being. Physical activity had a strong relationship with well-being (*beta* = 4.41), following gender (*beta* = −5.60) and followed by resilience (*beta* = 4.37) and number of diseases (*beta* = −2.35). Age did not present any statistically significant relationship with SPWB.

Model 2b, which included resilience and cultural activity as independent variables, explained 33.45% of the variance in well-being. Cultural activity had the strongest relationship with well-being (*beta* = 8.23), followed by gender (*beta* = −6.09), resilience (*beta* = 4.25), and number of diseases (*beta* = −2.24). Age did not show any statistically significant relationship with SPWB in this model either.

### 3.5. Mediation Models

In our population, we found a statistically significant relationship between physical and cultural activity, on the one hand, and both resilience and well-being, on the other. Therefore, we explored the possibility that resilience mediates, at least in part, the relationship of the two variables with SPWB. We verified this hypothesis through a mediation analysis approach. Considering a system with three variables (*X*, *Y* and *W*), all correlated with each other, this approach is aimed at answering the following questions: (1) how much of the association between *X* and *Y* can be explained through the effect of *X* on *W*, and sequentially on *Y*; and (2) is there still a direct association between *X* and *Y* after adjusting for the effects through *W*?

Two mediation models were tested: resilience mediating the relationship between physical activity and well-being, and resilience mediating the relationship between cultural activity and well-being. According to the Sobel test, the indirect effect of physical activity on well-being via resilience is significantly different from 0 (*Z* = 5.06, *p* < 0.001). Furthermore, the indirect effect of cultural activity on well-being via resilience is also significantly different from 0 (*Z* = 6.16, *p* < 0.001). This finding indicates that, at least in part, resilience mediates the association of both physical and cultural activity with well-being.

To estimate the ratio of mediated to total effect, we used the method described by Preacher and Kelley [48]. Following this approach, we found that the ratio of indirect to total effect for physical activity was 0.28 (95% *CI* = 0.18–0.40, *p* = 0.001), and likewise it was 0.28 for cultural activity (95% *CI* = 0.19–0.39, *p* = 0.001). 

Thus, resilience plays a significant role in the relationships between physical activity and well-being, as well as between cultural activity and well-being. In both cases, it seems to act as a partial mediator, accounting for about one third of the total effect of each variable on SPWB.

## 4. Discussion

Leisure activities, physical, cultural or social, contribute to resilience and well-being by helping to cope with stress. In this article, we have analyzed, in a sample of citizens of the metropolitan area of Naples, the relationship between leisure activities (both regular physical exercise and participation in cultural and social activities) on subjective well-being, and the role of resilience in this relationship.

Residents of the metropolitan area of Naples participating in this survey scored on average in the area of moderate distress according to Chassany et al. and Grossi et al. (SPWB 68.76 ± 19.18 on a scale 0–110) [38,39]. This SPWB score is by far lower than that reported by Grossi et al. for Northern (79.34 ± 17.71), Central (78.04 ± 17.12), and Southern (75.47 ± 17.91) Italy [39], and, even more alarming, is almost 12 points lower than that reported in 2013 for a sample of 1000 citizens of Milan (SPWB 82.14 ± 15.63) [41]. We cannot rule out that the low SPWB score of the sample population of residents of the metropolitan area of Naples reflects the detrimental effects of the economic and social crisis that has affected Naples since 2008. In particular, we must consider that Naples has not shown signs of socio-economic recovery, despite the fact that most cities and regions in the Eurozone are now finally performing beyond expectations, and thus the prospect of socio-economic stagnation remains unchallenged, with a likely negative impact on SPWB. 

To the best of our knowledge, evaluations of the resilience score of Naples residents were lacking so far. According to the CD-RISC2, the sample population analyzed here reported a resilience score of 5.6 ± 1.89. This score is lower than the 6.91 score reported in a general population survey of US adults [42], is close to the score of 5.85 ± 1.1 reported by Jeong et al. in a Korean study, and is higher than that observed (5.03 ± 1.37) by Ni et al. among the general population in Hong Kong [50,51]. Given the diversity of these societies in a global perspective, cultural factors and contexts may account for the differences in resilience score reported above. 

Another relevant aspect of our study is the role of cultural participation. According to Eurostat and Eurobarometer data [44,52], the percentage of people participating in cultural activities should be calculated on the basis of participation intensity, and thus only attendees with medium, high and very high participation levels should be considered. Within the context of our study, we consider how cultural participation impacts upon well-being, and, in line with the average cultural participation rates in Italy [52], we found that 51% of the residents of the metropolitan area of Naples participating in the survey present medium, high, or very high levels of attendance to social and cultural activities (data not shown). However, as noted above, since low-access attendees present a SPWB score higher than that of subjects not engaged in cultural and social activity, for the purpose of our study the percentage of participants rises to 82%. This result is not surprising, considering that in a comparable study on the city of Milan [41] only 6% of the subjects included in the survey did not participate in cultural events, raising the percentage of participants to 94% of the analyzed sample.

Moreover, we considered physical activity. The WHO estimates indicate that, in Europe, more than one third of adults and two thirds of adolescents are insufficiently active, and, according to the WHO physical activity fact sheet [53], one in four adults globally is not active enough. As for Italy, 33.3% of the citizens are engaged in physical activities, compared to a value of 23.3% for the Southern regions [54]. Interestingly, 38% of the subjects participating in our survey engaged in physical activity, a percentage higher than that reported above for the Southern regions. The importance of physical activity as both a preventive and therapeutic measure for several non-communicable diseases, like cardiovascular diseases, is a possible explanation for this observation. However, we cannot rule out that also self-aesthetic reasons and compliance to current body image standards may have played a role.

To investigate the relationship among the variables of interest, we have performed a correlation analysis. Not surprisingly, we found a statistically significant positive association between age and a number of diagnosed conditions, and a negative association between age and participation in cultural and social activities (*r* = −0.18, *p* < 0.001), as well as with physical activity (*r* = −0.19, *p* < 0.001). The inverse relation between leisure activity and age is well documented, and apparently indicates that a progressive disengagement from leisure activities is part of growing older. Although participation in leisure activities is important for physical and mental health, the negative association between both number of diagnosed conditions and participation in cultural and social activities (*r* = −0.25, *p* < 0.001) and physical activity (*r* = −0.22, *p* < 0.001) indicates that poor health (i.e., an increasing number of diseases) limits engagement in leisure activities. Consistently, the number of diagnosed diseases is negatively correlated with SPWB. Considering the close relationship between subjective psychological well-being and health, this connection becomes particularly important while aging, when the prevalence of chronic illness increases. In this respect, high levels of SPWB have been associated with a decreased risk in mortality in general [55,56]. Interestingly, in in our study sample, resilience did not correlate with number of diseases. 

In our sample population, the two dimensions of resilience apparently contribute differentially to the total Resilience score. On average, our group of subjects was more able to react to adversity (3.03 ± 1.03 Resilience score) than to adapt to change (2.7 ± 1.05 Resilience score), both *p* < 0.0001. A possibly telling explanation is that, after 3000 years of troubled history, the only way to survive was effectively reacting to adversity. Thus, Neapolitans developed special resilience skills, fixing resilience into a sort of “social steady state”, difficult to modify through contingent factors and events, and privileging the static component of resilience (“endure”) with respect to the dynamic one (“change”). At a superficial level, Neapolitan people are stereotyped as optimistic about the future despite the present, inclined to enjoy life and “take it easy” even when facing material scarcity and adversity, and with a nuanced nostalgia for a lost Golden Age [57,58]. However, below the surface, the Neapolitan collective psychology is characterized by a deep disenchantment, often turning into desperate irony and a tragic sense of life, that undoubtedly belongs to the ethos of the city. Magical thinking and religiosity mix in the effort to regain a sense of control upon uncertain events [57], not unlike what happens in cities characterized by similar mixes of functional and dysfunctional aspects such as Rio de Janeiro and New Orleans. The root of Neapolitan resilience is therefore to be sought in a blend of unconscious fear of powerful, uncontrollable forces and a strong determination to survive.

Within the sample analyzed, we found evidence of lower SPWB levels in females, confirming the SPWB gender differential in favor of males that is commonly found in the literature [41,59,60,61]. We also confirmed a strong positive association between resilience and SPWB [61,62,63,64,65]. Finally, both participation in social activities and physical activity were positively associated with resilience, as well as with SPWB. Since the seminal work of Konlaan et al. [66], the role of leisure activities in improving well-being has been widely explored. Apart from observational studies, intervention studies have also demonstrated the efficacy of leisure activities in the promotion of well-being [67,68,69]. Moreover, leisure helps to build up resilience, as a form of stress survival strategy [70]. The results reported above indicate a positive association of cultural, social, and physical activity with SPWB and resilience. Thus, we applied a multiple regression approach to separate the possible effects of cultural and social activities and physical activity on resilience. Leisure activities (Model 1a included physical activity as independent variable, and Model 1b included cultural and social activities as independent variable) explained a relatively low share of the total variance in resilience. These were, however, the independent variables most strongly related to resilience and the only variables reaching statistical significance. We then applied the same approach to investigate the relationship between leisure activities and SPWB. Model 2a, including physical activity and resilience as independent variables, explained 32.39% of the variance in SPWB. In particular, physical activity turned out to have a strong relationship with SPWB (*beta* = 4.41). Model 2b including cultural and social activities as independent variable explained 33.45% of variance in SPWB. In particular, we found that also cultural and social activities have a strong relationship with SPWB (*beta* = 8.23). Since our results show a statistically significant relationship between leisure activities (both physical and cultural-social) and SPWB and resilience, respectively, we further explored whether resilience would mediate, at least in part, the relation between a certain type of leisure activity and SPWB, and we tested our hypothesis using a “mediation analysis” approach. In particular, we tested two mediation models: (1) does resilience mediate the relationship between physical activity and well-being?; and (2) does resilience mediate the relationship between cultural and social activities and well-being?

Our results indicated that resilience plays a significant role in the relationship between physical activity and well-being, as well as between cultural and social activities and well-being. In both cases, resilience seems to act as a partial mediator, accounting for about one third of the total effect of each variable on SPWB. Quoting Diener et al.: “the primary function of leisure is to produce psychological detachment from work, duties and obligations, which is a precursor to the restoration of psychological and physical resources required for continued functioning and well-being” [71].

Engagement in leisure activities is a free choice that individuals can also modify and adjust during the course of their lives. In any event, leisure activities produce positive emotions, which can counteract the effects of negative emotions, as well as generate positive thoughts and experiences, which in turn can help people face adversity and bounce back from negative experiences. Appropriate leisure activities may accompany people throughout their life, and at all ages they may improve the resilience→well-being circuitry. Starting from Konlaan et al. [66], several lines of evidence, such as the Trøndelag Health Study (HUNT study) [72], show that receptive cultural attendance improves well-being and quality of life in non-mentally ill subjects. Cultural and social engagement may then qualify as preventive interventions at the population level. Participation in social and cultural activities favors social interaction and connection, stimulates curiosity and cognition, and provides a sense of existential fulfillment. In other words, participation in social and cultural activities stimulates dispositions that are conducive to various forms of human and social development which positively reflect into quality of life and subjective well-being, and this seems to be confirmed also for a relatively problematic urban environment such as Naples.

## 5. Limitations

Though our findings indicate that resilience plays a significant role in the relationship between physical activity and well-being, as well as between cultural activity and well-being, and though these results may have important implications for the well-being of adults of all ages, this study has several limitations. Self-report questionnaires enable the collection of a large amount of quantitative data, and generalization of the findings is possible, if the sample is randomly collected. Nevertheless, we are aware of the limitations of using self-report questionnaires, whose main disadvantage might be the possibility of providing invalid or biased answers. In particular, respondents may not answer truthfully because of social desirability, acquiescent and non-acquiescent response bias, and clarity of the items. However, some of the problems can be countered through the careful design and application of self-reporting measures. For example, response bias can be removed by ‘reversing’ half the questions on a questionnaire so that the variable is scored by positive responses on half the questions and negative responses on the other half, thus cancelling out any response bias. This is the approach applied in the SPWB questionnaire, where half of the question are reversed.

The cross-sectional design of the study limited our ability to infer direction of causality. A longitudinal design would better support the conclusion that resilience is in part responsible of the link between physical health and well-being, and between cultural activity and well-being [73,74,75]. However, this is not always true. In this regard, Pek and Hoyle note that in recent years there has sometimes been a superficial use of longitudinal design, and this did not allow to overcome the weaknesses of cross-section design [76]. Moreover, this problem is associated with the difficulties that arise when taking ongoing measurements on the same sample in order to prepare a longitudinal design. We are aware that the results of the mediation model in the current study should be interpreted with caution, and future research is needed to give a definite response.

## 6. Conclusions

Considering that welfare costs are one of the major sources of public finance deficits in the EU, and that health care systems all over Europe struggle to remain socially and financially sustainable, to find resource-effective tools to promote health and prevent diseases is a key priority. In this perspective, a “cultural welfare” approach, aiming at encouraging participation in social and cultural activities through suitable, targeted policies could represent a resource-effective salutogenesis tool [77].

Recent research by Tawakol et al. and Ben-Shaanan et al. [78,79] sheds new light upon the biological mechanisms connecting physical and positive mental health. This work indicates there is a route linking health to well-being and resilience in the brain-immune system axis, and in both cases the crucial step is the relief of stress or the enhancement of the reward circuitry. If to cure is an institutional responsibility of the health care system, to care is a responsibility of everyone. In this view, in the design of public health prevention and promotion interventions, a systemic approach is called for, drawing upon a wide, diverse yet closely integrated spectrum of expertise. Even in complex, and to some extent dysfunctional, urban environments such as Naples, thinking of leisure activities as a driver of health promotion and disease prevention may be effective. Moreover, the role of local culture and history in shaping the behavior of residents and in enhancing their capacity to cope with stressors through shared meaningful experiences and culturally specific forms of resilience should not be overlooked. The culture and history of places may matter for public health more than it is commonly thought, and as a consequence more attention should be paid to the specific environmental characteristics of heritage cities and their possible impact on residents’ attitudes toward, and preparedness to tackle, health-related social challenges. Our study shows how major aspects of urban culture, as crystallized in the resilience construct, may play a complex mediating role in the relationship between forms of human cultivation such as cultural, social and physical activities, and well-being. More learning and experimentation is called for to assess the full potential of such dimensions in inspiring innovative public health approaches.

## Figures and Tables

**Table 1:** Characteristics of the sample.

Total Number of Subjects	1182	
**Gender**		%
males	543	46
females	638	54
**AGE**		
all	55.73 ± 15.70	
males	57.15 ± 15.8	
females	54.53 ± 15.53	
>65	72.29 ± 6.139	32
<65	47.86 ± 12.39	68
**Civil status**		
Married	731	61.8
Divorced	30	2.5
Widows	81	6.9
Single	250	21.2
NA	90	7.6
**Education**		
Elementary	95	8.0
Junior High school	220	18.6
Senior High School	428	36.2
University	417	35.3
NA	22	1.9
**Occupation**		
Working	527	44.6
Unemployed	68 *	5.8
Retired	301	25.5
Student	52	4.4
Housewife	170	14.4
NA	63	5.3

* unemployement rate = 12.9.

**Table 2:** Correlations of demographic, health, leisure activities, and well-being variables.

	Age	Gender	Resilience	SPWB	Cultural Activity	Physical Activity
Gender ^a^	−0.08					
Resilience	−0.01	0.02				
SPWB	−0.09	−0.14 *	0.46 *			
Cultural activity	−0.18 *	0.06	0.19 *	0.28 *		
Physical activity	−0.19 *	−0.03	0.15 *	0.24 *	0.24 *	
N° of diseases	0.37 *	0.00	−0.06	−0.31 *	−0.25 *	−0.22 *

^a^ 0 = male, 1 = female. * = *p* < 0.001.

**Table 3:** Regression models with Resilience as the dependent variable: Model 1a (physical activity).

Coefficients	Estimate	Std.Error	t-Value	Pr(>|t|)
(Intercept)	5.171	0.230	22.481	<2 × 10^−16^ *
Age	0.004	0.004	1.089	0.276
Gender ^a^	0.121	0.110	1.1	0.271
Physical activity	0.5871	0.115	5.063	4.7 × 10^−7^ *
N° of diseases	−0.036	0.027	−1.329	0.184

Residual standard error: 1.87 on 1177 degrees of freedom; Multiple R-squared:0.02599, Adjusted R-squared:0.02268; F-statistic: 7.852 on 4 and 1177 DF, *p*-value: 3.028 × 10^−6^; ^a^ = 0 = male, 1 = female. * = *p* < 0.001.

**Table 4:** Regression models with Resilience as the dependent variable: Model 1b (cultural activity).

Coefficients	Estimate	Std.Error	t-Value	Pr(>|t|)
(Intercept)	4.597	0.265	17.324	<2 × 10^−16^ *
Age	0.004	0.004	1.036	0.3
Gender ^a^	0.059	0.109	0.543	0.587
Cultural activity	0.968	0.154	6.289	4.5 × 10^−10^ *
N° of diseases	−0.025	0.027	−0.906	0.365

Residual standard error: 1.859 on 1177 degrees of freedom; Multiple R-squared:0.03713, Adjusted R-squared:0.03386; F-statistic: 11.35 on 4 and 1177 DF, *p*-value: 4.887 × 10^−9^; ^a^ = 0 = male, 1 = female. * = *p* < 0.001.

**Table 5:** Regression models with self-reported psychological well-being (SPWB) as the dependent variable: Model 2a (physical activity).

Coefficients	Estimate	Std.Error	t Value	Pr(>|t|)	
(Intercept)	49.916	2.319	21.524	<2 × 10^−16^	*
Resilience	4.37	0.246	17.779	<2 × 10^−16^	*
Age	0.024	0.032	0.744	0.457	
Gender ^a^	−5.608	0.925	−6.062	1.81 × 10^−9^	*
Physical activity	4.418	0.989	4.47	8.59 × 10^−6^	*
N° of diseases	−2.355	0.231		<2 × 10^−16^	*

Residual standard error: 15.77 on 1176 degrees of freedom; Multiple R-squared: 0.3267, Adjusted R-squared:0.3239; F-statistic: 114.1 on 5 and 1176 DF, *p*-value: < 2.2 × 10^−16^; ^a^ 0 = male, 1 = female. * = *p* < 0.001.

**Table 6:** Regression models with self-reported psychological well-being (SPWB) as the dependent variable: Model 2b (cultural activity).

Coefficients	Estimate	Std.Error	t Value	Pr(>|t|)
(Intercept)	45.271	2.501	18.098	<2 × 10^−16^
Resilience	4.254	0.245	17.344	<2 × 10^−16^
Age	0.024	0.031	0.768	0.443
Gender ^a^	−6.099	0.919	−6.64	4.78 × 10^−11^
Cultural activity	8.237	1.316	6.257	5.48 × 10^−10^
N° of diseases	−2.24	0.231	−9.695	<2 × 10^−16^

Residual standard error: 15.64 on 1176 degrees of freedom; Multiple R-squared: 0.3374, Adjusted R-squared:0.3345; F-statistic: 119.7 on 5 and 1176 DF, *p*-value: < 2.2 × 10^−16^; ^a^ 0 = male, 1 = female.
